# Organizational change and the risk of sickness absence: a longitudinal multilevel analysis of organizational unit-level change in hospitals

**DOI:** 10.1186/s12913-019-4745-2

**Published:** 2019-11-27

**Authors:** Anniken Grønstad, Lars Erik Kjekshus, Trond Tjerbo, Vilde Hoff Bernstrøm

**Affiliations:** 1 Department of Health Management and Health Economics, Institute of Health and Society, Faculty of Medicine, University of Oslo, Forskningsveien 3A, N-0373 Oslo, Norway; 2 Department of Sociology and Human Geography, Faculty of Social Sciences, University of Oslo, Moltke Moes vei 31, N-0851 Oslo, Norway; 3 Work Research Institute, OsloMet, Oslo Metropolitan University, Stensberggata 26, N-0170 Oslo, Norway

**Keywords:** Organizational change, Sickness absence, Healthcare, Hospitals, Norway, Longitudinal multilevel logistic regression

## Abstract

**Background:**

Organizational change is often associated with reduced employee health and increased sickness absence. However, most studies in the field accentuate major organizational change and often do not distinguish between and compare types of change. The aim of this study was to examine the different relationships between six unit-level changes (upsizing, downsizing, merger, spin-off, outsourcing and insourcing) and sickness absence among hospital employees.

**Methods:**

The study population included employees working in a large Norwegian hospital (*n* = 26,252). Data on unit-level changes and employee sickness absence were retrieved from objective hospital registers for the period January 2011 to December 2016. The odds of entering short- (< = 8 days) and long-term (> = 9 days) sickness absence for each individual employee were estimated in a longitudinal multilevel random effects logistic regression model.

**Results:**

Unit-level organizational change was associated with both increasing and decreasing odds of short-term sickness absence compared to stability, but the direction depended on the type and stages of change. The odds of long-term sickness absence significantly decreased in relation to unit-level upsizing and unit-level outsourcing.

**Conclusions:**

The results from this study suggested that certain types of change, such as unit-level downsizing, may produce greater strain and concerns among employees, possibly contributing to an increased risk of sickness absence at certain stages of the change. By contrast, changes such as unit-level insourcing and unit-level upsizing were related to decreased odds of sickness absence, possibly due to positive change characteristics.

## Background

Hospitals worldwide operate in an ever-changing environment. The remodeling of healthcare organizations seeks to produce fruitful avenues for innovation and growth that benefit both patients and society [1]. However, organizational change is often associated with adverse employee health and increased sickness absence [2–4]. Earlier research has documented increasing risks of employee sickness absence and adverse health effects following organization-level downsizing [5], merger [6], expansion [7] and restructuring [8]. Nevertheless, some researchers have found a weak decrease, or no increase at all, in sickness absence after similar changes [9–11]. Previous research on organizational change and sickness absence has often refrained from differentiating between and comparing various types of change [12] and focused largely on organization-level changes rather than minor change initiatives [13]. As a result, less is known about the potential adverse health effects of different types of unit-level organizational changes [14]. Generating more information on the relationship between unit-level organizational change and employee sickness absence is essential because such changes occur more frequently in organizational life [15]. What is more, distinguishing between and comparing types of change by measuring their individual influence on sickness absence is important to account for the possibility that changes may influence employees and the work environment differently [16]. Researchers have often theoretically argued that organizational change may lead to reduced health and increased sickness absence by fostering a work environment often described as strained and possibly harmful to employee health [17, 18]. Certain studies have also tested this assumption. Kivimäki et al. [19] found that the combination of multiple mechanisms including reduced job control, increased job demands and higher job insecurity explained 49% of the increase in sickness absence relating to organizational downsizing. In a similar vein, the results of a recent multilevel study suggested a possible mediating role of job demands, job control and social support on the relationship between different changes (i.e. reorganization, downsizing and layoffs) and increased mental distress [20].

Although several studies have highlighted potentially undesirable health outcomes relating to organizational change, it is unlikely that all changes are experienced in a similar manner. For example, while adverse health effects of downsizing may partially be explained by reduced job security, it is unlikely that the same mechanism occurs in relation to upsizing. Instead, employing more personnel may strengthen employees’ perception of job security. As such, organizational change may also nurture positive work-related experiences possibly protecting workers from ailments and promoting employee health [21]. Indeed, earlier research has also demonstrated associations between desirable changes and lowered strain [22], improved job satisfaction [23] and decreased long-term sickness absence [24]. Therefore, investigating the different relationships between each type of unit-level change and sickness absence remains important.

While most studies have focused on large-scale changes, there are a few articles investigating the effects of unit-level organizational change on employee sickness absence. Ingelsrud [25] observed a positive relationship between changes in hospital units and long-term sickness absence. Similarly, Bernstrøm and Kjekshus [15], on the same dataset, studied structural unit-level and patient-related changes and found a significantly elevated risk of long-term sickness absence following structural changes only, suggesting that the two types of change influenced employee sickness absence differently. However, these changes were aggregated at the organizational level and the different kinds of structural changes were not studied individually. Recently, a Danish study examined the effects of different types of unit-level change on employee sickness absence and observed statistically significant relationships between mergers and layoffs and increased long-term sickness absence [26].

The aim of the present study was to distinguish between six types of organizational change occurring at the unit-level of a major hospital: upsizing, downsizing, merger, spin-off, outsourcing and insourcing, and measure each change type’s correlation with individual-level employee sickness absence*.* To this end, the research questions were: Are different types of unit-level organizational change related to employee short- and long-term sickness absence? If so, in what direction?

Based on previous literature, we expected that unit-level organizational change would correlate with increased sickness absence. However, by distinguishing between types of change, we also expected that the relationship between unit-level organizational change and sickness absence would not be the same for all unit-level change types.

### Setting the context of change: change rationale, economic and employment situation

The association between work and employee health may be influenced by contextual factors [11, 27–30]. Therefore, to better understand the relationship between unit-level change and sickness absence, we also need to consider the context in which it proceeds. In that regard, the present study was situated in a large Norwegian hospital in the period 2011–2016. Since the 1990s, Norwegian hospitals have been subjected to several major changes. At the beginning of the millennium, the central government acquired ownership of all Norwegian public hospitals that had previously been under county government administration. As a consequence, hospitals were largely exposed to organizational change in the following years. In the period 2000–2010, > 90% of public hospitals were exposed to at least one merger [6, 31, 32]. Specifically, a merger between two regional health enterprises (RHEs) took place in 2007. Two years later, the hospital in this study similarly underwent a major change involving the merging of four hospitals located in the capital region. In the subsequent years, numerous additional changes have been implemented subjecting the hospital to high-paced change. Following the hospital-merger, city districts previously belonging to the hospital in this study were reassigned to a neighboring hospital. Consequently, there was a need to reduce personnel and initiate other types of change to realign the hospital structure to the new and reduced patient pool.

The wider context of the present study was characterized by financial prosperity and a strong employment situation with little risk of long-term unemployment. During the study period, the unemployment rate was low at the level of 3–4% [33]. Considering employment prospects, the Norwegian healthcare sector was and is increasingly in demand of personnel, particularly nurses, due to the challenges associated with an aging population and diminishing labour supply [34, 35]. Accordingly, the context of the present study was partly in contrast to several earlier studies investigating organizational change and sickness absence in times of economic decline [5, 7, 27]. Moreover, downsizing in Norwegian hospitals rarely involves dismissals. Instead, excess employees are often offered employment and are channeled into work elsewhere within the public sector [36].

Finally, Norwegian legislation and the Norwegian welfare system are designed to protect employees and reduce the financial burden of sickness absence and redundancy. Accordingly, employees are entitled to 100% salary reimbursement for both short- and long-term sickness absence (with a maximum duration of 52 weeks), and approximately 62% salary reimbursement in cases of unemployment (with a maximum duration of 104 weeks) [37, 38]. These financial benefits contribute to making the Norwegian welfare system one of the most generous in the world. In addition to egalitarian sickness and unemployment benefits, the Norwegian labour market is characterized by robust employment protection legislation. Indeed, the Work Environment Act dictates that it is illegal to terminate employees’ contracts due to sickness absence lasting less than 1 year [39]. Regarding contract type, OECD statistics [40] show that Norway operates with a slightly higher level of protection against contract termination among those who are permanently employed, compared to the OECD average. Additionally, compared with other countries, Norway is at the top in terms of regulating the use of temporary employment [40].

## Methods

### Participants

#### The hospital

The study used data collected at a major Norwegian hospital during the period January 2011 to December 2016. The hospital has a workforce of more than 20,000 professionals working in several different geographical locations. The hospital’s main purpose is to provide high-quality healthcare. Additionally, the hospital has a research center and is responsible for teaching and training medical personnel.

#### The employees

The hospital records included 29,863 unique employee ID numbers in the given study period. To ensure sufficient exposure to unit-level change, the analyses included fulltime employees only, limiting the sample to those with a minimum of 80% cumulative employment positions. To ensure that the units subject to change were real work units, they were restricted to those having employed a minimum of four employees at one time. The final sample consisted of 26,252 employees, 1349 units and 965 departments. The participants investigated in this study were part of a heterogeneous workforce in terms of age (mean age 41, SD 12) and occupation. Indeed, while medical doctors (15%) and nurses (33%) constitute the largest employee groups, the sample also included other health professionals, kitchen and facility services, economists, HR personnel, engineers and IT professionals, in addition to professionals in other fields. However, the employees were predominantly female (73%).

The average annual salary for hospital employees was NOK 540,000 (approximately € 56,000). Absence levels in our sample were comparable to the overall sector average. National absence statistics show that approximately 6% of employees had a physician certified absence spell on specific reference dates during the study period. For health services, and in our sample, the corresponding amount of physician certified absence, is approximately 7% [41]. During an entire quarter, approximately 39% of the employees entered a period of short-term sickness absence, and 11% of the employees entered a period of long-term sickness absence.

### Data

Data were retrieved from the hospital’s human resources department (HR) and included merged registries built on scrambled employee ID numbers. By employing the hospital’s HR registers for unit-level change and sickness absence, it was possible to track and isolate exposure to unit-level changes and employee sickness absence for the same individual employees over time. The registers were continuously logged by the hospital, but for the purpose of analyses, the data were aggregated into employee-quarters. The analytical model of the study was longitudinal and measured organizational change and employee sickness absence quarterly between January 2011 and December 2016.

#### Sickness absence

The data on employee sickness absence were obtained from hospital registers. All sickness absence documentations, regardless of who issued them (i.e. self-certified or by the GP), must be forwarded to the hospital for recording. Each absence period was continuously registered by the hospital with a start and end date and the percentage of absence. Such recording was necessary in order for the hospital to provide substitute personnel for absent employees. The objective registers could therefore be considered highly accurate and complete. The analyses included all sickness absence periods irrespective of percentage. The data were checked for irregularities, in particular consecutive or coinciding sickness absence periods. In cases where absence periods were consecutive or coinciding, they were merged. In this study, periods of sickness absence were separated into short and long periods depending on whether employees were able to complete their own medical sick leave certificates [42]. Short absence periods were operationalized as < = 8 days and long spells were defined as > = 9 days because hospital employees are only required to submit a medical sick leave certificate from the GP for absences lasting > = 9 days. For each quarter, we registered whether the employee had an episode of sickness absence (1) or not (0). Short- and long-term sickness absences were analyzed separately.

#### Organizational change

The data on organizational change were acquired from hospital records on employment contracts. These data contained historical information on employees’ contracts for the period January 2011 to December 2016. The organizational change measurement was created by tracking the units in which the employees work for each quarter, and their transfers between work-units. In the present study, organizational change was operationalized as six different internal unit-level changes, namely: upsizing, downsizing, merger, spin-off, outsourcing and insourcing, in addition to stability acting as the control group. Please see Table 1 for a description and operationalization of the change variables applied in this study. The change variables were dichotomous, given values of 0 (stability) or 1 (unit-level change). For each quarter, we registered whether the employee had experienced a specific unit-level change, and each of the six changes were analyzed and compared to stability. For each quarter, the employee might be coded as expecting a specific change during the upcoming quarter (e.g. unit-level merger in the next quarter), currently undergoing a specific change (e.g. unit-level merger this quarter), having undergone a specific change previous quarter (e.g. unit-level merger in the previous quarter) or experiencing stability. The different operationalizations of change types employed in this study were mutually exclusive so that the employees could not be coded as experiencing more than one change in a given quarter. However, the employees might undergo multiple changes during the six-year study period in the hospital. Furthermore, the stability-control quarter covered any quarter without a change. Each employee would therefore generally have multiple quarters of stability occurring both prior to unit-level change and after unit- level change.

**Table 1:** Operationalization and description of unit-level organizational changes

Change type	Criterion/criteria	Description/example
Merger	At least one of the following criteria must be met: • If at least 90% of employees at one unit (A) move to a different unit (B), all employees at the old unit (A) are categorized as having been through a unit-level merger. • If at least 20% of employees at the new unit (B) come from a merger unit – all employees at the new unit (B) are also categorized as having been through a unit-level merger. • If a new unit is created (B), with at least 90% of employees coming from at least two different units in the hospital - all employees in the new unit are also categorized as having experienced a unit-level merger.	Two units become one. For example, two wards treating blood related diseases at the hospital in this study merged both physically and operationally as they were decided to continue their operations in one hospital location.
Downsizing	At least one of the following criteria must be met: • A unit reduces the staff by at least 20% during the quarter and the unit is not categorized as experiencing unit-level merger, unit-level outsourcing or unit-level insourcing. • An employee is categorized as experiencing a unit-level downsizing if there has been a downsizing in the unit the employee works in at the beginning of and/or at the end of the quarter.	A reduction in the number of personnel. To define downsizing quantitatively, we applied the same level as Røed and Fevang [36], who defined major downsizing as a reduction exceeding 20%. Similar to downsizing at the organizational level, unit-level downsizing generally occurs without layoffs [36]. Strategies aimed at staff reductions in a unit entails withstanding renewal of temporary contracts, not filling vacant positions, in addition to transferring employees to other jobs within the hospital or to a neighboring hospital.
Spin-off	The following criterion must be met: • If a new unit is created (B), with at least 90% of employees coming from the same old unit (A) in the hospital, the employees in the new unit (B) are categorized as experiencing a unit-level spin-off.	A new organizational unit is created within the organization by making part of an old organizational unit an organizational work-unit in its own right. For example, the emergency psychiatric unit was separated within the hospital to become an independent unit.
Insourcing	The following criterion must be met: • If a new unit (B) is established with at least 90% of the employees coming from outside the hospital, then all the employees in the new unit (B) are categorized as experiencing a unit-level insourcing.	An organizational unit not previously a part of the hospital becomes an integral part. The hospital becomes responsible for the subject area, the employees and the day-to-day operations. Geographical relocation is not necessary. The area of forensic subjects was transferred from the Norwegian Institute of Public Health to the hospital in this study.
Upsizing	At least one of the following criteria must be met: • If the unit increases personnel by at least 20% during the quarter, but the unit is not categorized as experiencing unit-level merger, unit-level outsourcing or unit-level insourcing. • An employee is categorized as experiencing a unit-level upsizing if there has been an upsizing in the unit the employee works in at the beginning of and/or at the end of the quarter.	Increasing the number of personnel. In order to define upsizing quantitatively, we applied the same level as Røed and Fevang [36].
Outsourcing	The following criterion must be met: • If at least 90% of the employees in one unit (A) stop working in the organization – all employees at the old unit (A) are categorized as experiencing a unit-level outsourcing.	The units are relocated organizationally (e.g. to another hospital) and the unit is no longer a part of the hospital. A geographical relocation is not required. For example, employees in the purchasing department at the hospital in this study were outsourced to another employer with which the hospital had close affiliations. The department still serves the hospital staff, but they are organized and officially employed by an external “sister-organization”.
Stability	The following criterion must be met: • If the unit experiences none of the changes above. This implies that at least 80% of the employees working in the unit during the previous quarter still work in the unit at the end of the current quarter, and that at least 80% of the employees working in the unit at the end of the quarter also worked in the unit during the previous quarter. It also suggests that the unit has not grown or been reduced in size by 20% or more.	

Three change variables that comprised very few employees necessitate further explanation. The variable “unit-level insourcing next quarter” captured only eight hospital employees who would begin working in a unit subject to insourcing during the next quarter. Similarly, “unit-level outsourcing this quarter” and “unit-level outsourcing previous quarter” captured eight and 22 employees, respectively who remained in an outsourced unit. Consequently, these change variables provided limited information and meaning for the purpose of this study and we have therefore not presented them in our results.

#### Control variables

In this paper, control variables included job position, multiple jobs, temporary contracts, age, salary and gender. Job position was coded into physician, nurse (control), other patient-oriented position, administration/management, kitchen/cleaning/orderly, other operations (e.g. IT), and other. Some of the employees included in the study sample had more than one job at the hospital. These jobs, when combined, amount to the minimum of an 80% position (e.g. 2 × 40% contracts). To control for having more than one job, the variable “multiple job holder” was included in the analyses. Furthermore, increases in sickness absence in relation to organizational change have been shown to relate to permanent employees, but not to temporary workers [43, 44]. As the use of temporary contracts is common in Norwegian hospitals (32% of the hospital employees in this study had temporary contracts), the variable “temporary contract” was included to control for its potential influence on the unit-level change−/ sickness absence relationship. Age was measured in years and salary comprised the contracted remuneration. Gender was dummy coded (female =1; male =0).

### Statistical analysis

Differences in spells of sickness absence were analyzed using multilevel logistic regression with random intercept taking into account the dependency in the data (i.e. clustering effects). Our outcome variable (sickness absence) was measured at the lowest level of analysis, employee-quarters. We included three random intercepts; for employees, for units and for departments. Hence, in our statistical model, employee quarters were nested within employees, employees were nested within units and units were nested within departments. A multilevel design was considered suitable for the analysis of clustered data because it permits relaxation of the assumption that observations must be statistically independent by inherently generating estimates that account for the non-independence [45, 46]. Data are considered clustered when observations within the same cluster are expected to be related to each other [47, 48].

The threshold for statistical significance was set to *P* < .01. We investigated the relationships between six different types of unit-level change, at three different time points, and with two different measures of sickness absence. The nature of multiple testing increases the risk of finding significant effects by chance [49]. Therefore, to account for the possibility of type I error (false positives), one should consider the threshold for statistical significance. Consequently, we adopted a stricter threshold for statistical significance of *P* < .01, compared to the more commonly used level of *P* < .05. All statistical analyses were performed using the melogit command in STATA/SE, version 15.1 (StataCorp LP, College Station, TX, USA, http:www.stata.com/company/contract/).

## Results

The distribution of employees experiencing each of the different types of change is presented in Table 2. The most prevalent unit-level changes occurring in the hospital during the study period were upsizing and downsizing of units.

**Table 2:** Distribution of employees experiencing different types of change

Unit-level change	No. of employees undergoing unit-level change	% of n (26,252)
Upsizing	9629	37%
Downsizing	9230	35%
Merger	2148	8%
Spin-off	885	3%
Outsourcing	388	1%
Insourcing	82	< 1%

The results are shown in two models. Model 1 reveals the association between unit-level change and sickness absence prior to the inclusion of control variables, and Model 2 displays the association between unit-level change and sickness absence after including control variables. Overall, the inclusion of control variables produced minor differences in results. However, one correlation was shown to be insignificant: the relationship between unit-level downsizing next quarter and long-term sickness absence. Given the limited difference between the two models, emphasis is placed on Model 2 (with control variables) for the interpretation of the results. By focusing on the results including control variables, the probability of the results reflecting spurious correlations due to differences in demographic variables (e.g. if sickness absence increases due to fewer employees on temporary contracts after change) is reduced (Table 3).

**Table 3:** Unit-level organizational change and sickness absence – multilevel random effects analyses

Change type	Model 1^a^				Model 2^b^			
	Short-term SA		Long-term SA		Short-term SA		Long-term SA	
	(<=8 days)		(>=9 days)		(<=8 days)		(>=9 days)	
	OR	95% CI	OR	95% CI	OR	95% CI	OR	95% CI
Unit-level upsizing next quarter	1.01	(0.95–1.07) ns	1.001	(0.92–1.09) ns	1.02	(0.96–1.08) ns	1.02	(0.94–1.12) ns
Unit-level upsizing this quarter	0.75	(0.72–0.79) ^***^	0.83	(0.78–0.89) ^***^	0.77	(0.73–0.80) ^***^	0.89	(0.83–0.95) ^**^
Unit-level upsizing previous quarter	0.83	(0.80–0.87) ^***^	0.83	(0.77–0.89) ^***^	0.85	(0.81–0.89) ^***^	0.88	(0.82–0.94) ^***^
Unit-level downsizing next quarter	0.65	(0.62–0.68) ^***^	0.92	(0.86–0.98) ^**^	0.67	(0.64–0.69) ^***^	0.96	(0.91–1.03) ns
Unit-level downsizing this quarter	1.11	(1.06–1.16) ^***^	0.92	(0.86–0.98) ns	1.11	(1.06–1.16) ^***^	0.94	(0.88–1.01) ns
Unit-level downsizing previous quarter	1.20	(1.14–1.27) ^***^	1.01	(0.93–1.09) ns	1.22	(1.15–1.29) ^***^	1.04	(0.96–1.13) ns
Unit-level merger next quarter	0.87	(0.77–0.99) ns	0.86	(0.72–1.03) ns	0.86	(0.76–0.97) ns	0.89	(0.74–1.06) ns
Unit-level merger this quarter	1.12	(1.01–1.24) ns	1.10	(0.96–1.27) ns	1.11	(1.01–1.23) ns	1.14	(0.99–1.31) ns
Unit-level merger previous quarter	0.97	(0.88–1.07) ns	0.92	(0.79–1.06) ns	0.97	(0.87–1.07) ns	0.95	(0.82–1.10) ns
Unit-level spin-off next quarter	0.94	(0.78–1.13) ns	0.87	(0.67–1.14) ns	0.94	(0.78–1.12) ns	0.89	(0.68–1.15) ns
Unit-level spin-off this quarter	1.09	(0.93–1.27) ns	1.007	(0.80–1.25) ns	1.08	(0.92–1.26) ns	1.01	(0.80–1.26) ns
Unit-level spin-off previous quarter	1.05	(0.89–1.23) ns	0.72	(0.56–0.93) ns	1.04	(0.88–1.22) ns	0.72	(0.56–0.93) ns
Unit-level outsourcing next quarter	0.37	(0.27–0.51) ^***^	0.49	(0.30–0.80) ^**^	0.36	(0.27–0.50) ^***^	0.51	(0.32–0.83) ^**^
Unit-level insourcing this quarter	0.17	(0.07–0.42) ^***^	0.44 ^c^	(0.12–1.51) ns	0.15	(0.06–0.37) ^***^	0.44	(0.13–1.50) ns
Unit-level insourcing previous quarter	0.48	(0.29–0.79) ^**^	0.40 ^c^	(0.15–1.03) ns	0.46	(0.28–0.75) ^**^	0.46	(0.18–1.19) ns

Multilevel random effects logistic regression with short- and long-term sickness absence as outcome variables. Employee-quarters nested within employees, employees nested within units and units nested within departments

Significance: the level of statistical significance was set to *p* < .01. In the table, ^**^ = *p* < .01; ^***^ = *p* < .001

*Abbreviations*: *SA* sickness absence, *OR* odds ratio, *CI* 95% confidence intervals, *ns* not significant

N Upsizing 24,688 (313,056 observations); N Downsizing 24,838 (314,053); N Merger 24,165 (296,376); N Spin-off 24,084 (294,466); N Outsourcing 24,094 (293,352); N Insourcing 24,093 (293,310)

^a^ Control variables not included

^b^ Control variables included

^c^ The results are calculated with the restriction “iterate (10)” to make converging possible with three random intercepts. The results are similar to those with two random intercepts (employee and work-unit) and without the iterate restriction

Employees experiencing unit-level upsizing had a significantly reduced risk of both short- and long-term sickness absence in the change quarter (OR = 0.77; 95% CI = 0.73–0.80 and OR = 0.89; 95% CI = 0.83–0.95, respectively) and in the subsequent quarter (OR = 0.85; 95% CI = 0.81–0.89 and OR = 0.88; 95% CI =0.82–0.94, respectively). Experiencing unit-level downsizing was significantly associated with lowered odds of short-term sickness absence in the quarter prior to change, compared to stability (OR = 0.67; 95% CI = 0.64–0.69). The results further revealed that unit-level downsizing correlated significantly with increased risk of short-term sickness absence in the change quarter (OR = 1.11; 95% CI = 1.06–1.16) and in the following quarter, compared to stability (OR = 1.22; 95% CI = 1.15–1.29). Similar to unit-level downsizing, the results for unit-level merger demonstrated decreasing odds of short-term sickness absence prior to the change (OR = 0.86; 95% CI = 0.76–0.97) and increasing odds of short-term sickness absence in the change quarter (OR = 1.11; 95% CI = 1.01–1.23). However, these results did not reach statistical significance at the *P* < .01 level. As with unit-level merger, the relationship between unit-level spin-off in the previous quarter and reduced long-term sickness absence (OR = 0.72; 95% CI = 0.56–0.93) showed no significant effect at the *P* < .01 level. For unit-level outsourcing, the results showed that the likelihood of both short- and long-term sickness absence dropped significantly in the quarter prior to the change (OR = 0.36; 95% CI = 0.27–0.50 and OR = 0.51; 95% CI = 0.32–0.83, respectively). Finally, experiencing unit-level insourcing was significantly associated with lowered odds of short-term sickness absence both during (OR = 0.15; 95% CI = 0.06–0.37) and after change (OR = 0.46; 95% CI = 0.28–0.75).

## Discussion

The present study investigated the relationship between six different unit-level organizational changes and employee sickness absence before, during and after unit-level change. A main finding was that the relationship between unit-level change and employee sickness absence is complex and varies depending on change type and stages of change.

Unit-level upsizing, the type of change experienced by most employees in the hospital, was associated with reduced risk of both short- and long-term sickness absence during and after the change. These results contradicted our expectations that unit-level organizational change is associated with increased sickness absence. However, the results corroborate previous research observing declining levels of sickness absence after upsizing and expansion [11, 50] and they supported our assumption that different types of organizational change relate differently to employee sickness absence. Lowered sickness absence levels pertaining to this particular type of change may reflect positive employee appraisals of change [51]. Indeed, increasing the number of personnel may contribute to reduced demands because the amount of work is shared and allocated among more employees. Such positive consequences might contribute to and explain some of the reduced risk of sickness absence associated with this type of change.

However, it is important to note that these results also contradict one previous study that identified accumulated exposure to major expansion as a significant risk factor for long-term sickness absence [7]. Different contexts in which upsizing unfolds could be helpful in explaining why employees experiencing similar changes, react differently. While Westerlund et al. [7] found support for increased long-term sickness absence relating to rapid expansion in times of a financial recession, our result of reduced sickness absence relating to upsizing was obtained in a time of financial prosperity. Accordingly, the absence of societal instability due to a financial crisis may leave more room for planning change in advance, thereby allowing both employer and employees to prepare and create readiness for change [52, 53]. Consequently, we may expect that upsizing, in our study, is more likely to be associated with work dimensions such as strengthened job security and a positive work environment - factors one indeed would expect to be related to upsizing [7].

The results pertaining to unit-level downsizing showed that the risk of short-term sickness absence significantly increased during and after change, and significantly declined in the quarter preceding the change, compared to stability. The largest increase in sickness absence was observed in the quarter following unit-level downsizing as the odds of short-term sickness absence increased by 22%. Given that 39% of the employees entered at least one period of short-term sickness absence during the quarter, a 22% increase in odds entailed an increased risk of approximately five additional employees per 100 employees on short-term sickness absence during the quarter. However, in logistic regression, effect sizes should be interpreted with caution [54].

These results showing increases in sickness absence during and after change are congruent with earlier research on organizational-level downsizing [19] and unit-level changes [15, 25, 26]. Unlike these studies, however, we found increased risk of short-term sickness absence rather than long-term sickness absence. Salient features associated with this type of change such as increased demands and a reduced sense of control [3, 43] may explain the increased risk of short-term sickness absence.

However, the results also indicated that the odds of sickness absence declined prior to unit-level downsizing. In line with the Whitehall II results, our findings showed that employees often react to change before it is implemented [55]. However, while Whitehall II showed deterioration of self-reported health prior to organizational change, our results showed reduced sickness absence. The direction of our results (i.e. reduced sickness absence) may be explained by perceptions of job insecurity. In Norwegian hospitals, employees are rarely subjected to layoffs, even during organizational change. However, the broad use of temporary contracts may leave employees worried that their contracts may not be renewed. Hence, insecurity regarding prospective employment may leave temporary employees more eager to go to work despite illness, thereby possibly lowering the level of sickness absence and contributing to an increased risk of sickness presenteeism [43, 56, 57].

We did not find significant effects at the *P* < .01 significance level for the relationships between unit-level merger and unit-level spin-off and sickness absence. However, absence of evidence is not necessarily evidence of absence [58]. This is particularly important to note in the event of unit-level merger, which would have been significant at the *P* < .05 level. Merger constitutes a type of change that is often implemented in the Norwegian healthcare sector [6], and has previously been related to elevated long-term sickness absence [26].

Experiencing unit-level outsourcing and unit-level insourcing was associated with a reduced risk of short-term sickness absence and the odds of long-term sickness absence declined in the quarter prior to unit-level outsourcing. The largest decrease in sickness absence was observed in the quarter during unit-level insourcing as the odds of short-term sickness absence dropped by 83%, amounting to approximately 30 fewer employees per 100 employees on short-term sickness absence during the quarter. In the quarter following unit-level insourcing, the odds of short-term sickness absence were 52% lower, compared to stability, indicating that approximately 16 fewer employees per 100 employees were on short-term sickness absence during the quarter.

As with the results pertaining to unit-level upsizing, these results did not support our expectations of a positive relationship between unit-level organizational change and sickness absence, but they provide support for our assumption that different types of organizational change relate differently to employee sickness absence. To explain decreases in sickness absence in relation to these types of change, one might emphasize their potential to elicit positive employee experiences. While previous research often focused on the potential for all changes to produce strain and potential illness [3], recent research finds declining levels of sickness absence following specific types of change (e.g. job change) [24]. Based on these findings, we therefore argue that it is important to emphasize potential positive, health promoting aspects of unit-level change. For example, it is possible that a unit-level insourcing enables closer and more rewarding collaborations among employees. Moreover, unit-level outsourcing, in this hospital, may be carried out in a way that counteracts potential strain and hazard by strengthening the employees’ perceptions of job security and justice [59]. Such positive consequences might contribute to and explain some of the reduced risk of sickness absence associated with these types of change.

### Organizational change and the Norwegian context

As we discussed in the introduction, the relationship between organizational change and sickness absence should be interpreted in the context in which it occurs. Given the rarity of layoffs in Norwegian hospitals [36], combined with a generous social security system, a healthy economic climate and solid employment protection, we may speculate that any adverse health effects of organizational change on the employees would be eased within a Norwegian context. However, our result that sickness absence still rises following certain types of unit-level change, may indicate that such features remain insufficient to eradicate change-related strain among Norwegian hospital employees. Furthermore, job insecurity is not necessarily limited to concerns about unemployment but may also involve the perceived loss of valued circumstances at work. Indeed, studies have revealed that restructuring not involving layoffs may also profoundly and adversely influence employee well-being, weaken co-worker relationships, and reduce organizational commitment [60, 61], and that it may increase the likelihood of nurses resorting to sickness absence [36].

### Practical implications for health services

For healthcare managers and practitioners, increased awareness and knowledge about the types of organizational change that generate increased expenses due to employee sickness absence remain important. For example, during a unit-level downsizing, the odds of entering a period of short-term sickness absence increased by 11%. Given that 39% of the employees were registered as beginning at least one instance of short-term sickness absence during the quarter, an 11% increase in odds equaled three additional employees per 100 employees on short-term sickness absence during the quarter. However, it is equally important for healthcare managers and practitioners to be aware that certain types of change were associated with reduced risk of employee sickness absence. Although more knowledge is necessary in order to better understand why the risk of sickness absence differs depending on types of change, two main practical implications are suggested based on the findings of this study. First, by identifying the types of change that were related to increased sickness absence, healthcare managers and practitioners are able to develop tailored goal-oriented action plans in advance to limit the adversity and strain associated with these types of change. Second, by identifying types of change that correlated with reduced sickness absence, such as unit-level insourcing and unit-level upsizing, healthcare managers and practitioners are able to pinpoint and reinforce change characteristics that are potentially health-protecting and health-promoting such as ensuring a better allocation of workload and facilitating improved teamwork.

### Strengths and limitations

The present study investigated the impact of unit-level change on employee sickness absence longitudinally. An important strength of this paper was that it combined data on organizational change and sickness absence retrieved from hospital registers. These data were not biased by individual perceptions and thereby facilitated the measurement of dependent and independent variables objectively over time, with marginal missing data. In addition, the longitudinal multilevel method enabled the study to examine individual correlations between unit-level change and employee sickness absence before, during and after unit-level change, compared to stability. Longitudinal studies measuring the same individuals repeatedly over time are considered a methodological advantage [19]. A drawback of the study was that the unbiased sources of data did not offer any information on potential mechanisms influencing the association between employee unit-level change and employee sickness absence. It is possible that additional data on employee perceptions might return more nuanced results. Future research should consider including employee experiences as potential mediating or moderating variables in the analyses.

Multiple statistical testing is more prone to type I error (false positives). To counter the risk of chance findings and increase the probability of appropriately rejecting the null hypothesis, we adopted a criterion of *P* < .01 [49, 62]. It is important to note, however, that the relationships between unit-level merger in the next quarter and in this quarter and short-term sickness absence had *P* values between .05 and .01.

The organizational change measurement was constructed based on employee data. While objective data is a great strength, we also considered such data a limitation because we could not rule out the possibility of misclassification when coding the changes (e.g. if the majority of employees in a unit voluntarily resigned in a given quarter without being replaced in the same quarter, the unit may appear to be downsized in that specific quarter). However, some types of unit-level change were easily identified and understood in the data, such as outsourcing (e.g. the entire unit was relocated simultaneously). Other types of unit-level change, such as downsizing, may include components of other types of unit-level change (e.g. a smaller, unidentified merger). Furthermore, we did not measure employee perceptions of unit-level change. Therefore, it is also possible that employees experienced some of the unit-level changes that we have coded as one change as multiple changes.

## Conclusion

The results from this study suggested that the risk of sickness absence differed depending on change type and stages of change. Certain types of change, such as unit-level downsizing, may potentially inflict greater strain and stimulate concerns among employees. This may in turn contribute to an increased risk of sickness absence during and after the changes. By contrast, changes such as unit-level insourcing and unit-level upsizing constitute types of change that may be beneficial for employees because they might contain positive characteristics such as encouraging and promoting a better distribution and allocation of workload, while at the same time possibly reinforcing employee perceptions of job security. Because these results have the potential to influence the risk of employee sickness absence differently, they have important practical implications for healthcare managers and policy makers when deciding what kinds of change to implement.

## Data Availability

The data upon which the research was conducted belong to the participating hospital and are therefore not publicly accessible. Upon reasonable request, the corresponding author can facilitate contact with representatives from the participating hospital.

## Abbreviations

CI: Confidence interval
GP: General Practitioner
HR: Human Resources
ID: Identification
IT: Information Technology
NOK: Norwegian kroner
NS: Not significant
NSD: Norwegian Social Science Data Service
OECD: Organisation for Economic Co-operation and Development
OR: Odds ratio
RHE: Regional health enterprise
SA: Sickness absence
SD: Standard deviation
